# Regulated Expressions of MMP-2, -9 by Diterpenoids from *Euphorbia formosana* Hayata

**DOI:** 10.3390/molecules17022082

**Published:** 2012-02-21

**Authors:** Chia-Chun Yu, Ching-Ruey Hsieh, George Hsiao, Pi-Yu Chen, Meng-Lun Chang, Hwa-Wen Yin, Tzong-Huei Lee, Ching-Kuo Lee

**Affiliations:** 1 Graduate Institute of Pharmacognosy, Taipei Medical University, 250 Wu Xin Street, Taipei 110, Taiwan; Email: orchidbutterfly@hotmail.com (C.-C.Y.); chang76819@yahoo.com.tw (M.-L.C.); 2 Taipei Medical University Hospital, Taipei Medical University, 252 Wu Xin Street, Taipei 110, Taiwan; Email: tmc761038@tmu.edu.tw; 3 Department of Pharmacology, Taipei Medical University, 250 Wu Xin Street, Taipei 110, Taiwan; Email: geohsiao@tmu.edu.tw; 4 School of Pharmacy, Taipei Medical University, 250 Wu Xin Street, Taipei 110, Taiwan;Email: e023089103@tmu.edu.tw; 5 Taiwan Forestry Research Institute, No. 53, Nan-Hai Road, Taipei, 100, Taiwan;Email: hwyin@tfri.gov.tw

**Keywords:** *Euphorbia formosana*, abietane diterpene, MMP-2, -9, HT-1080

## Abstract

Two new abietane type diterpenoids, namely *seco*-helioscopinolide (**1**) and 3β,7β-dihydroxy-*ent*-abieta-8,13-diene-12,16-olide (**2**) were isolated from the aerial parts of *Euphorbia formosana* Hayata together with helioscopinolide A (**3**), helioscopinolide B (**4**), helioscopinolide C (**5**) and *ent*-(5β,8α,9β,10α,12α)-12-hydroxyatis-16-ene-3,14-dione (**6**). The structures of compounds **1**−**6** were elucidated by analyzing their spectroscopic data and comparison with the literature. Further biological tests by gelatin zymographic analysis revealed that **3**−**5** significantly up-regulated the expressions and activation of MMP-2 and -9 in human fibrosarcoma cell line HT1080.

## 1. Introduction

**Figure 1:**
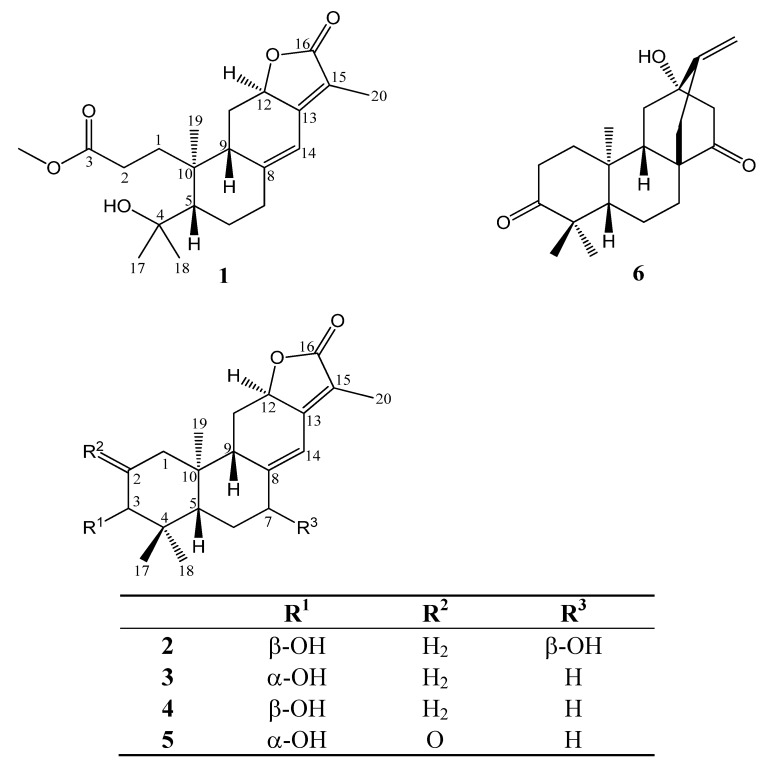
Chemical structures of compound **1**–**6**.

Increasing ultraviolet exposure of skin leads to acute and chronic detrimental cutaneous effects, which may result in the development of skin malignancies and photoaging [1]. Matrix metalloproteinases (MMPs) were known not only to exert functions in the resolution phase of wound healing but influence other wound-healing responses, such as inflammatory and re-epithelization [2]. Therefore, the screening of bioactive compounds from natural resources which can modulate the activity of MMPs, should prove worthwhile. *Euphorbia formosana* Hayata has long been used in folk medicine for the treatment of snakebite as well as many other dermatoses in Taiwan [3]. In this paper we describe the isolation and identification of a series of *ent*-abietane-type diterpenoids **1**–**6** (Figure 1) from *E. formosana*. During the past few years analogues of **1**–**6** were found to exhibit cytotoxic [4], antimicrobial [5], spasmolytic [6], antioxidant [7] and gastroprotective activities [8]. The bioactivities of **1**–**6** on MMP-2 and -9 in HT-1080 cells were evaluated in this study.

## 2. Results and Discussion

The aerial parts of *E. formosana* were extracted initially using methanol, and further partitioned between CHCl_3_ and H_2_O. The CHCl_3_ layer was then subjected to gravity silica column separation followed by HPLC purification to give two new diterpenoids **1** and **2**, along with helioscopinolide A (**3**) [9], helioscopinolide B (**4**) [10], helioscopinolide C (**5**) [11] and *ent*-(5β,8α,9β,10α,12α)-12-hydroxyatis-16-ene-3,14-dione (**6**) [12].

Compound **1** was isolated as colorless oil, and its molecular formula, C_21_H_30_O_5_, was established through analysis of its ^13^C-NMR and HR-ESI-MS data. The IR spectrum of **1** exhibited the presence of a hydroxyl group (3470 cm^−1^), a carbonyl group (1729 cm^−1^) and a double bond (1665 cm^−1^). The ^1^H-NMR data (Table 1) of **1** revealed signals for four methyl groups at δ_H_ 1.04 (s, H_3_-19), 1.23 (s, H_3_-17), 1.29 (s, H_3_-18) and 1.81 (s, H_3_-20), five methylene groups at δ_Η_ 1.50 (m, H-11a), 1.52 (m, H-6a), 1.78 (m, H-6b), 1.86 (m, H-1a), 2.12 (m, H-7a), 2.17 (m, H-2a), 2.44 (td, *J* = 3.3, 13.1 Hz, H-7b), 2.55 (dd, *J* = 5.8, 13.5 Hz, H-11b), 2.60 (ddd, *J* = 4.6, 11.1, 13.5 Hz, H-2b) and 2.75 (ddd, *J* = 4.6, 11.1, 13.2 Hz, H-1b), four methine protons at δ_Η_ 1.69 (dd, *J* = 2.7, 12.4 Hz, H-5), 2.33 (d, *J* = 8.4 Hz, H-9), 4.88 (dd, *J* = 6.1, 13.5 Hz, H-12) and 6.26 (s, H-14) and one methoxyl proton at 3.66 (s, H_3_-21).

**Table 1:** ^1^H-NMR data of **1** and **2** [in CDCl_3_, 500 MHz, δ in ppm (mult., *J* in Hz)].

No.	1			2	
	^13^C	^1^H		^13^C	^1^H
**1a**	33.1 t	1.86 (m)		32.9 t	1.80 (m)
**1b**		2.75 (ddd, 4.6, 11.1, 13.2)			1.98 (m)
**2a**	28.8 t	2.17 (m)		26.9 t	1.62 (m)
**2b**		2.60 (ddd, 4.6, 11.1, 13.5)			1.96 (m)
**3**	174.8 s			75.6 d	3.40 (br s)
**4**	75.2 s			38.1 s	
**5**	51.8 d	1.69 (dd, 2.7, 12.4)		41.2 d	2.33 (dd, 2.5, 13.5)
**6a**	27.4 t	1.52 (m)		31.7 t	1.82 (td, 2.5, 13.5)
**6b**		1.78 (m)			1.59 (m)
**7a**	36.8 t	2.12 (m)		72.3 d	4.41 (br s)
**7b**		2.44 (td, 3.3, 13.1)			
**8**	151.7 s			153.8 s	
**9**	45.2 d	2.33 (d, 8.4)		47.4 d	2.82 (br d, 9.0)
**10**	45.5 s			42.4 s	
**11a**	27.4 t	1.50 (m)		28.2 t	1.37 (dt, 9.0, 13.5)
**11b**		2.55 (dd, 6.0, 13.5)			2.57 (dd, 6.5, 13.5)
**12**	75.9 d	4.88 (dd, 6.0, 13.5)		76.7 d	4.89 (dd, 6.5, 13.5)
**13**	155.9 s			156.7 s	
**14**	113.9 d	6.26 (s)		115.8 d	6.52 (br s)
**15**	116.6 s			118.4 s	
**16**	175.2 s			174.9 s	
**17**	27.6 q	1.23 (s)		29.4 q	0.94 (s)
**18**	34.8 q	1.29 (s)		22.8 q	0.86 (s)
**19**	20.4 q	1.04 (s)		16.6 q	0.96 (s)
**20**	8.3 q	1.81 (s)		8.5 q	1.78 (d, 1.5)
**21**	51.7 q	3.66 (s)			

**Figure 2:**
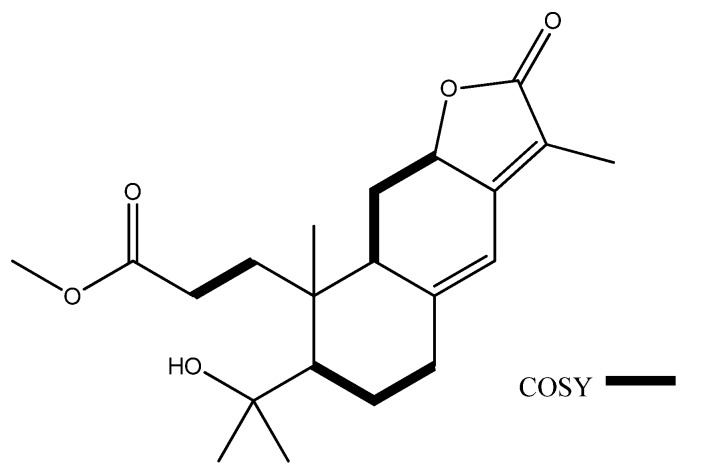
COSY of **1**.

The ^13^C-NMR spectrum coupled with DEPT analysis displayed 21 signals including four methyl carbons at δ_C_ 8.3 (C-20), 20.4 (C-19), 27.6 (C-17) and 34.8 (C-18), five methylene carbons at δ_C_ 27.4 (C-6 and -11), 28.8 (C-2), 33.1 (C-1) and 36.8 (C-7), four methine carbons at δ_C_ 45.2 (C-9), 51.8 (C-5), 75.9 (C-12) and 113.9 (C-14), seven quaternary carbons at δ_C_ 45.5 (C-10), 75.2 (C-4), 116.6 (C-15), 151.7 (C-8), 155.9 (C-13), 174.8 (C-3) and 175.2 (C-16), and one methoxyl carbon at δ_C_ 51.7 (C-21) (Table 1). Among the quaternary carbons, resonances at δ(C) 174.8 (C-3) and 175.2 (C-16) were assigned as two ester carbonyl groups. On account of the molecular formula, C_21_H_30_O_5_, since the index of hydrogen deficiency of **1** was seven, including two ester carbonyls and two olefinic functionalities, thus there should be three rings in **1**. Analysis of COSY (Figure 2) and HSQC spectral data allowed the assignments of three spin systems including two aliphatic chains, (–H-5–H_2_-6–H_2_-7–) and (–H-9–H_2_-11–H-12–), one two-resonance unit, (–H_2_-1–H_2_-2–), and five germinal-coupled methylene functionalities, (H_2_-1, H_2_-2, H_2_-6, H_2_-7 and H_2_–11).

**Figure 3:**
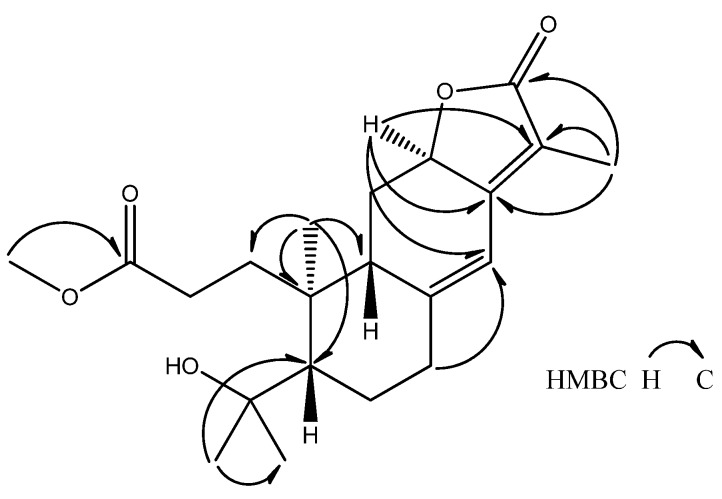
Selected HMBC of **1**.

In the HMBC spectrum of **1** (Figure 3), key long range proton-carbon correlations including H-7/C-14, H-12/C-13, -14 and -15, H_3_-19/C-1, -5, -9 and -10, H_3_-17/C-5 and -18, H_3_-20/C-13, -15 and -16 and H_3_-21/C-3 coupled by above interpretations established that **1** was quite similar to helioscopinolide A (**3**), except for an opened A ring, a methoxyl group attached at C-3 and a hydroxyl group borne by its C-4. Accordingly, the structure of **1** was elucidated to be as shown (Figure 1) and was named *seco*-helioscopinolide. The methyl ester at C-3 of **1** was not an artifact on the methanolic extraction process, as evidenced by further HPLC analyses of an acetonic extract of the original materials.

Compound **2**, obtained as amorphous white powder, was deduced to have a molecular formula of C_20_H_28_O_4_ by its HR-ESI-MS and ^13^C-NMR. The IR spectrum of **2** revealed the presence of a hydroxyl group (3411 cm^−1^), a γ-lactone carbonyl (1731 cm^−1^) and a double bond (1667 cm^−1^). The ^1^H-NMR data of **2** revealed signals compatible with those of helioscopinolide B (**4**), except that a carbinol group was present at C-7 in **2** (Table 1). The ^1^H-NMR differences between **2** and **4** were also reflected in their ^13^C-NMR, in which δ_C_ 37.0 of C-7 in **4** downfield shifted to δ_C_ 72.3 in **2**. The structure of **2** was thus determined to be 7-hydroxyhelioscopinolide B. The relative configuration of both H-3 and H-7 was determined to be equatorial, due to their distinctive small coupling constants and chemical shifts (Table 1), which were further confirmed by comparison with its H-3 axial-oriented isomer, 3α,7β-dihydroxy-*ent*-abieta-8,13-diene-12,16-olide [4]. The chemical shifts of H-3 in **2** (δ_H-3_ 3.40) and its isomer (δ_H-3_ 3.25) also corroborated that their H-3s were equatorial- and axial-oriented, respectively, as judged by the anisotropic effect. Compound **2** adopted an *ent*-abietane-type diterpenoid skeleton as evidenced from a positive Cotton effect at 250 nm and a negative Cotton effect at 215 nm in the circular dichroism spectrum of **2** and comparison with the literature [13]. After considering all the spectroscopic data of **2**, its structure was thus elucidated as shown in Figure 1, and was named 3β,7β-dihydroxy-*ent*-abieta-8,13-diene-12,16-olide.

**Figure 4:**
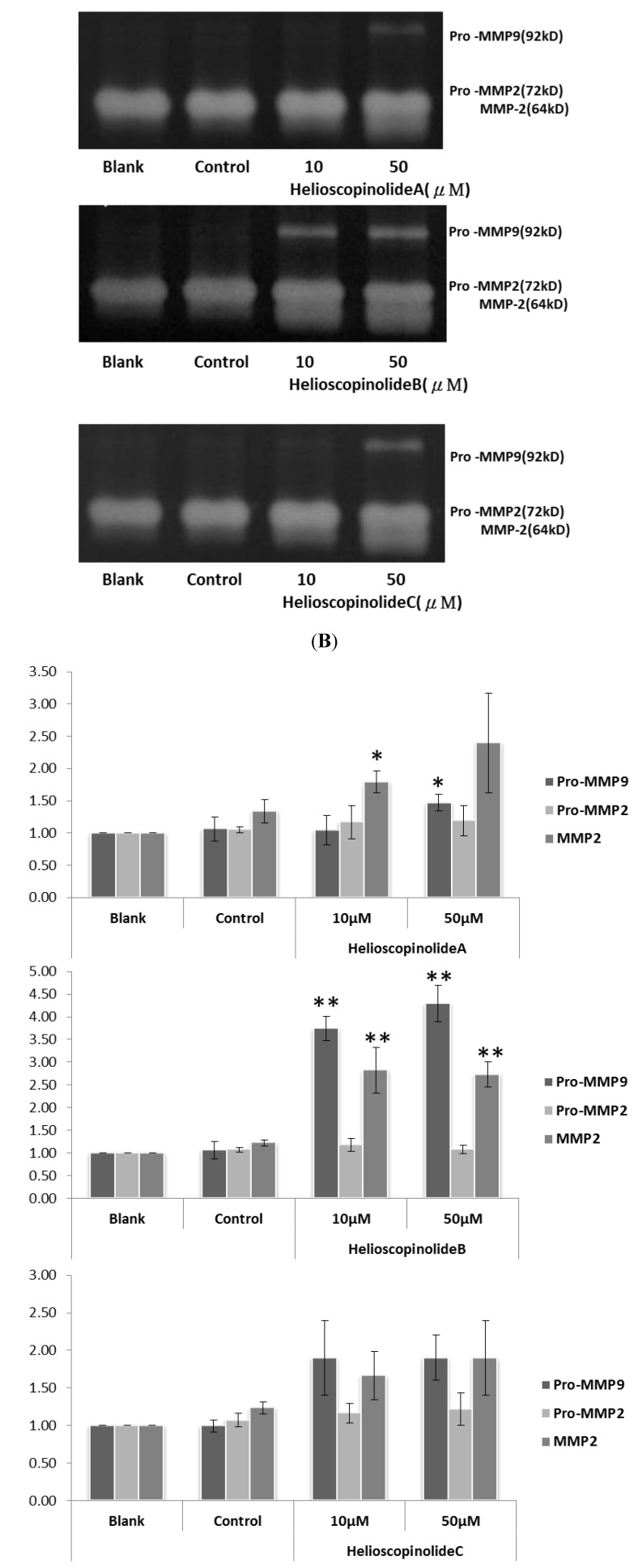
Effects of **3**–**5** on MMP-2, -9 activities. (A1-3) Results of gelatin zymography. Lane 1: blank; Lane 2: control; Lane 3-4: different concentration of compounds **3**–**5** (B1-3) Activities of MMP-2, -9 expressed as multiplication product of zone area and average gray value. Data represent the mean ± SD (n = 3). * *p* < 0.05, ** *p* < 0.01.

The MMP-2 and -9 modulating activity of the pure isolated compounds **1**−**6** in human fibrosarcoma cell line HT1080 were compared at the concentrations of 10 and 50 μM with blank group (Figure 4A). Among them, 3-hydroxyl *ent*-abietane compounds **3**-**5** exhibited significantly up-regulated the expressions of MMP-2, -9 according to gelatin zymography analysis (Figure 4B). The MMPs activity is regulated by transcriptional and post-transcriptional levels. The post-transcriptional activation of MMPs requires specific conditions, such as proteolytic removal of the propeptide, disruption of the bond between the cysteine sulhydryl moiety, and even their dephosphorylation [14,15] The intensive mode of action of **3**–**5** on posttranscriptional MMPs should remain to be further investigated.

## 3. Experimental

### 3.1. General

Optical rotations were measured on a JASCO P-1020 digital polarimeter (Tokyo, Japan). ^1^H- and ^13^C-NMR were acquired on a Bruker DMX-500 SB spectrometer (Ettlingen, Germany). High resolution and low resolution mass spectra were obtained using a Waters LCT Premier XE (Waters, Manchester, UK) and ABI API 4000 Q-TRAP (Foster City, CA, USA), respectively. IR spectra were recorded on a JASCO FT/IR 4100 spectrometer (Tokyo, Japan). UV and CD spectra were measured on a Thermo Helios α spectrophotometer (Waltham, MA, USA) and JASCO J-720 circular dichroism spectrometer (Tokyo, Japan), respectively. Silica gel (40–63 μm, Merck, Darmstadt, Germany) was used for gravity column chromatography. Pre-coated silica gel plates (Si 60 F_254_, 0.2 mm, Merck, Darmstadt, Germany) were used for analytical TLC. HPLC was performed using a semi-preparative column (Hibar^®^ Fertigäute, 10 × 250 mm, Merck).

### 3.2. Plant Material

Aerial parts of *Euphorbia formosana* Hayata was collected from Changhua County in July, 2004 and was identified by Dr. S. Y. Chen in the Council of Agriculture, Executive Yuan. Voucher Specimens (No. CKL07012004) have been deposited in the School of Pharmacy, Taipei Medical University, Taipei, Taiwan.

### 3.3. Extraction and Isolation

The dried aerial parts (11.8 kg) of *E. formosana* was extracted three times with MeOH (100 L), then partitioned between CHCl_3_ and water (2 L, 1:1, v/v). Subsequently, the dried CHCl_3_ layer (300 g) was pre-absorbed using 400 g silica gel (70–230 mesh), applied onto a 4,950 g silica gel open column (230–400 mesh) and then eluted by mixtures of *n*-hexane, ethyl acetate and methanol in a stepwise gradient mode. Each fraction collected (500 mL) was checked by TLC, and dipping in 10% H_2_SO_4_ in ethanol were used in the detection of compounds. Subsequently, 348 fractions were combined into 15 portions. Portion #10 was purified by HPLC on a semi-preparative normal-phase column with *n*-hexane/ethyl acetate/acetone/dichloromethane (25:6:4:10, v/v/v/v) as eluent, 3 mL/min, afforded **3** (9.0 mg). The same portion was purified using the same HPLC column with *n*-hexane/acetone (5:1, v/v) as eluent, 3 mL/min, gave **1** (9.6 mg), **4** (8.4 mg), **5** (7.8 mg) and **6** (4.5 mg). The portion #15 was purified using the same HPLC column with *n*-hexane/acetone (7:3, v/v) as eluent, 3 mL/min, obtained **2** (22.5 mg).

### 3.4. seco-Helioscopinolide *(**1**)*

Colorless oil. [α]^25^_D_ = +50.8 (*c* = 0.1, CHCl_3_). UV (MeOH): 276 (3.9). IR (KBr): 3470 (−OH), 2928, 2877, 1729 (C=O), 1665 (C=C), 1438, 1024. ^1^H-NMR and ^13^C-NMR: see Table 1. ESI-MS: 363 [M+H]^+^. HR-ESI-MS: 363.2181 ([M+H]^+^, C_21_H_31_O_5_, calc.363.2171).

### 3.5. 3β,7β-Dihydroxy-ent-abieta-8,13-diene-16,12-olide *(**2**)*

Amorphous white powder. [α]^25^_D_ = +180.4 (*c* = 0.1, MeOH). UV (MeOH): 272 (4.0). IR (KBr): 3411 (−OH), 2927, 1731 (C=O), 1667 (C=C), 1451, 1385, 1301, 1168, 1090, 1030. ^1^H-NMR and ^13^C-NMR: see Table 1. ESI-MS: 333 [M+H]^+^. HR-ESI-MS: 331.1905 ([M−H]^–^, C_20_H_27_O_4_, calc. 331.1909).

### 3.6. Cell Culture

HT1080 human fibrosarcoma cells which were from American Type Culture Collection (ATCC: CCL-121) were cultured in RPMI-1640 medium (Gibco) supplemented with 10% fetal bovine serum (Gibco), 100 U/mL penicillin, 100 mg/mL streptomycin. Cultures were maintained in a humidified incubator at 37 °C in 5% CO_2_/95% air.

### 3.7. Gelatin Zymography

Gelatin zymography was used to determine expression and activities of MMP-2 and -9 [16]. HT1080 cell line were seeded in 24-well plates using serum free medium for 24hr cell adhesion and growth. After 48 h of test compounds with different concentrations incubation, conditioned medium was collected to analyzed the activities of MMP-2 and -9.

## 4. Conclusions

In this report, we have identified six ent-abietane-type diterpenoids including two new ones from Euphorbia formosana Hayata. Ent-abietane-type diterpenoids such as compounds **3**–**5** significantly up-regulated the expressions and activation of MMP-2 and -9 in human fibrosarcoma cell line HT1080, and could potentially be leads for the development of novel wound-healing drugs.
